# Hepatic Arachidonic Acid Dysregulation Impairs Double‐Negative Regulatory T Cell‐Mediated Immunoregulation via CD39 Downregulation and Drives MASLD Development

**DOI:** 10.1155/jimr/6950401

**Published:** 2026-06-25

**Authors:** Hua Jin, Xiyu Wang, Xinjuan Liu, Dong Zhang, Guangyong Sun

**Affiliations:** ^1^ Medical Research Center, Beijing Institute of Respiratory Medicine and Beijing Chao-Yang Hospital, Capital Medical University, Beijing, 100020, China, ccmu.edu.cn; ^2^ Department of Gastroenterology, Beijing Chao-Yang Hospital, Capital Medical University, Beijing, 100020, China, ccmu.edu.cn

**Keywords:** CD39, double-negative regulatory t cells, immunoregulatory function, MASLD

## Abstract

Immune dysregulation is a key driver of metabolic dysfunction‐associated steatotic liver disease (MASLD). Double‐negative regulatory T (DNT) cells, which are essential for hepatic immune homeostasis, are functionally impaired in MASLD. CD39, an ectoenzyme that converts ATP to adenosine, sustains DNT cell viability and immunoregulatory function. However, the mechanisms of MASLD‐induced CD39 dysregulation remain unknown. In this study, a transcriptomic analysis of hepatic DNT cells from MASLD mice revealed reduced ATP hydrolysis, as confirmed by flow cytometry, and lower CD39 level compared to normal control diet‐fed mice. Similarly, single‐cell transcriptomic data showed that CD39 was downregulated in hepatic DNT cells from MASLD patients, and higher CD39 expression was associated with enhanced cytotoxicity and viability. Furthermore, *in vitro* experiments confirmed that CD39 maintained the immunosuppressive function of DNT cells by hydrolyzing ATP and suppressing monocyte activation. Mechanistically, arachidonic acid (AA), which is enriched in the livers of MASLD, suppressed CD39 via hypoxia‐inducible factor‐1α (HIF‐1α). This AA‐HIF‐1α‐CD39 axis impaired DNT cell‐mediated monocyte immunosuppression, accelerating immune imbalance and MASLD progression. These findings establish the AA‐HIF‐1α‐CD39 axis as a key pathway driving DNT cell dysfunction in MASLD, highlighting potential therapeutic strategies targeting metabolic–immune crosstalk to restore hepatic immune balance.

## 1. Introduction

Metabolic dysfunction‐associated steatotic liver disease (MASLD), a global health burden driven by metabolic dysregulation and sustained lipid overload, is characterized by hepatic steatosis, inflammation, and fibrosis [1, 2]. Both innate and adaptive immune dysregulation have been implicated as critical drivers of MASLD pathogenesis [3]. Double‐negative regulatory T (DNT) cells, defined by the TCRαβ^+^CD4^−^CD8^−^ phenotype with the additional absence of NK1.1 in mice and the absence of CD56 in humans, serve as critical immunoregulatory T cells (Tregs) essential for maintaining immune homeostasis [4]. Our previous studies demonstrated functional DNT cell impairment in MASLD, including reduced cellular viability and diminished immunosuppressive capacity, whereas the adoptive transfer of healthy DNT cells significantly attenuated disease progression in murine models [5, 6]. However, the mechanisms underlying DNT cell dysfunction in MASLD remain unclear.

The purinergic signaling pathway, which is mediated by ectonucleoside triphosphate diphosphohydrolase‐1 (CD39), is critical for maintaining immune homeostasis in inflammatory diseases. CD39 has been identified as a surface marker of Tregs, where it enzymatically converts extracellular ATP into adenosine to suppress proinflammatory effector T‐cell responses via A2A receptor‐mediated immunosuppressive signaling [7]. Notably, our study revealed that CD39 is also highly expressed in DNT cells and sustains DNT cell survival by converting toxic extracellular ATP into immunoregulatory adenosine. This enzymatic activity not only mitigates ATP‐induced cell death but also promotes the adenosine‐mediated upregulation of NKG2D expression, thereby enhancing the DNT cell immunoregulatory function [8]. However, it remains unclear whether MASLD disrupts this CD39‐dependent purinergic signaling axis in DNT cells or through what molecular mechanisms such dysregulation occurs.

Herein, we demonstrated that hepatic DNT cells in MASLD exhibited a markedly reduced CD39 expression. DNT cells with high CD39 expression exhibited enhanced immunosuppressive function and viability, suggesting that downregulation of CD39 may contribute to DNT cell dysfunction in MASLD. Mechanistically, MASLD‐associated lipid overload, particularly arachidonic acid (AA), suppressed CD39 expression in DNT cells via hypoxia‐inducible factor‐1α (HIF‐1α). This study identified the AA‐HIF‐1α‐CD39 axis as a key driver of DNT cell dysfunction, offering novel therapeutic targets to restore the immune balance in MASLD.

## 2. Methods

### 2.1. Mice and MASLD Model Establishment

Male C57BL/6 (wild‐type [WT]) mice were purchased from the Beijing Vital River Laboratory (Beijing, China), and *Cd39* ^−/−^ mice were provided by Dr. Simon C. Robson. The MASLD model was established by feeding mice a methionine–choline‐deficient (MCD; Beijing HFK Bioscience, 10401, Beijing, China) diet for 4 weeks, a choline‐deficient high‐fat diet (CDHFD; Research Diets, D05010402, NJ, USA) for 16 weeks, or a high‐fat diet (HFD; Research Diets, D12492) for 16 weeks. The mice in the control group were fed a normal control diet (NCD) for the same duration. For HIF‐1α inhibition experiments, mice fed the MCD diet for 14 days were subsequently treated with the HIF‐1α inhibitor LW6 (10 mg/kg; HY‐13671, MCE) by intraperitoneal injection every other day for an additional 14 days, while control mice received vehicle treatment. The mice were housed at Beijing Chao‐Yang Hospital in a specific pathogen‐free environment with a controlled temperature and a 12‐h light/dark cycle. All animal experiments were conducted in accordance with ethical guidelines and approved by the Animal Ethics Committee of Beijing Chao‐Yang Hospital (Approval Number 24‐2007).

### 2.2. Flow Cytometry Analysis

Hepatic immune cells were harvested via enzymatic digestion, and 33% Percoll density gradient separation was performed, as previously reported [9]. The cells were stained with surface markers using the following fluorochrome‐conjugated anti‐mouse antibodies: anti‐CD45 (clone I3/2.3), anti‐CD3 (17A2), anti‐NK1.1 (S17016D), anti‐TCRβ (H57‐597), anti‐CD4 (GK1.5), anti‐CD8a (53‐6.7), and anti‐CD39 (Duha59). For intracellular staining, the cells were fixed with fixation buffer (420801, BioLegend) and subsequently stained with either anti‐granzyme B (QA16A02) or anti‐TNF‐α (MP6‐XT22). For nuclear antigen staining, the cells were fixed and permeabilized using the Transcription Factor Fixation/Permeabilization Buffer Set (424401, BioLegend), followed by intracellular staining with an anti‐HIF‐1α antibody (ab216842, Abcam) and goat anti‐rabbit IgG H&L (Alexa Fluor 488, ab150077, Abcam).

### 2.3. DNT Cell Expansion and *In Vitro* Cellular Assays

Single‐cell suspensions from mouse spleens and lymph nodes were subjected to red blood cell lysis. The cells were then incubated with biotin‐conjugated anti‐mouse CD4, CD8a, NK1.1, TCRγδ, TER119, CD19, and CD11b antibodies, followed by negative selection using streptavidin nanobeads (480016, BioLegend) to purify DNT cells. The isolated DNT cells were expanded in vitro for 7 days in the presence of recombinant mouse IL‐2 and mature dendritic cells. For stimulation assays, DNT cells were treated with AA (10, 25, or 50 µM), adrenic acid (ADA; 25 µM), or palmitic acid (PA; 25 µM) for 48 h, and cell viability as well as CD39 expression were analyzed by flow cytometry. To evaluate the role of ATP and adenosine signaling in DNT cell immunoregulation, WT and *Cd39* ^−^/^−^ DNT cells were treated with ATP (400 µM) with or without adenosine (400 µM), and NKG2D expression was analyzed by flow cytometry. For the inhibitor experiment, DNT cells were pretreated with the HIF‐1α inhibitor LW6 (10 µM) for 1 h, followed by stimulation with AA (25 µM) for 48 h, after which the CD39 expression was evaluated.

### 2.4. DNT Cells and Monocyte Coculture Assay

WT DNT, *Cd39* ^−/−^ DNT, or AA‐stimulated (25 µM, 48 h) DNT cells were cocultured with CD45.1 congenic mouse‐derived monocytes at a 1:2 ratio (DNT cells:monocytes) in the presence of 400 µM ATP with or without 400 µM adenosine. After 6 h, apoptosis and TNF‐α secretion in CD45.1^+^ monocytes were assessed by flow cytometry.

### 2.5. Statistical Analysis

All the data were expressed as the mean ± standard deviation and analyzed using GraphPad Prism 10.0 (GraphPad Software). The normality of the distribution was assessed using the Shapiro–Wilk test. Comparisons between two groups were performed with an unpaired Student’s *t*‐test for normally distributed data or the Mann–Whitney *U* test for nonnormally distributed data. Multiple group comparisons were conducted using one‐way ANOVA, followed by Sidak’s test for parametric data or the Kruskal–Wallis test with Dunn’s test for nonparametric data. Statistical significance was defined as *p* < 0.05.

## 3. Results

### 3.1. CD39 Expression is Downregulated in DNT Cells During MASLD Progression

**Figure 1:**
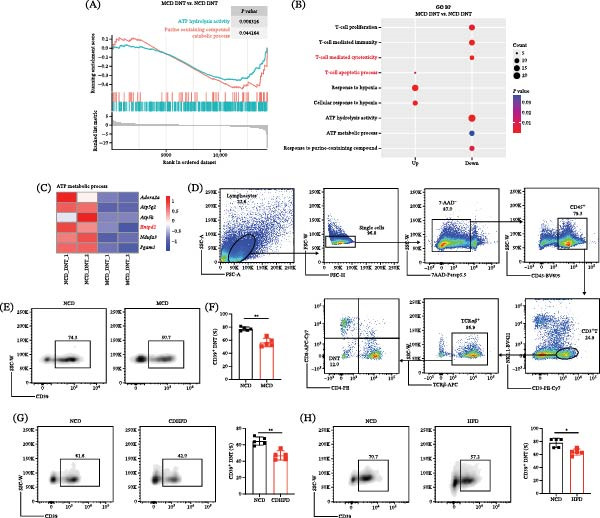
CD39 expression is downregulated in DNT cells during MASLD progression. (A) Gene set enrichment analysis (GSEA) was conducted on hepatic DNT cell transcriptomic data from methionine–choline‐deficient (MCD) diet‐induced MASLD mice and normal control diet (NCD)‐fed control mice. *n* = 2 biologically independent mice per group. (B) Gene ontology biological process (GO BP) analysis was performed using differentially expressed genes (DEGs, |fold change| ≥ 2, *p* < 0.05) between DNT cells isolated from MASLD model mice and control mice. (C) Heatmap displaying genes associated with ATP metabolic processes. (D) Flow cytometry gating strategy for DNT cells (CD45^+^CD3^+^NK1.1^−^TCRαβ^+^CD4^−^ CD8^−^) in liver. (E–H) Proportions of CD39^+^ DNT cells in the liver of mice fed with NCD, MCD, CDHFD, or HFD. Actual *p* values for panels F–H are 0.0002, 0.0008, and 0.0159. Error bars represent mean ± SD. *n* = 5 biologically independent mice per group. Statistical analysis was performed using an unpaired two‐tailed Student’s *t*‐test for panels F and G, and the Mann–Whitney *U* test for panel H.  ^∗^ *p* < 0.05,  ^∗∗^ *p* < 0.01.

To investigate alterations in purinergic signaling during MASLD progression, we analyzed our transcriptomic sequencing data of hepatic DNT cells from MCD diet‐induced MASLD mice and NCD‐fed control mice [5]. Compared with DNT cells derived from control mice, those derived from MCD‐fed mice presented lower ATP hydrolysis activity and purine‐containing compound catabolic process‐associated signaling (Figure 1A). Pathway enrichment analysis of differentially expressed genes (DEGs) revealed that MCD feeding significantly impaired ATP metabolism and hydrolysis activity in DNT cells. Additionally, MCD feeding led to a decrease in T‐cell‐mediated cytotoxicity and promoted T‐cell apoptosis (Figure 1B). In addition, we observed the significant downregulation of the expression of *Entpd1* (encoding CD39) and the adenosine receptor *Adora2a* in pathways associated with ATP metabolism (Figure 1C). Given the indispensable role of CD39‐mediated adenosine signaling in preserving DNT cell function, we hypothesized that MASLD progression disrupts this pathway, resulting in compromised immunoregulatory activity and viability deficits in DNT cells.

To further investigate CD39 expression dynamics in DNT cells during MASLD progression, we isolated hepatic immune cells via collagenase‐based enzymatic digestion and quantified CD39 levels in the DNT cell subset (CD45^+^CD3^+^NK1.1^−^ TCRαβ^+^CD4^−^CD8^−^) using flow cytometry (Figure 1D). The frequency of CD39‐positive DNT cells was significantly lower in the livers of the MCD diet‐fed mice than in the livers of the NCD‐fed mice (Figure 1E,F). Similarly, there was a significant decrease in CD39 expression on DNT cells in the MASLD models induced by the CDHFD and HFD, confirming the aforementioned phenomenon (Figure 1G,H).

### 3.2. Downregulated CD39 Expression in DNT Cells Impairs Immunoregulation to Promote MASLD

**Figure 2:**
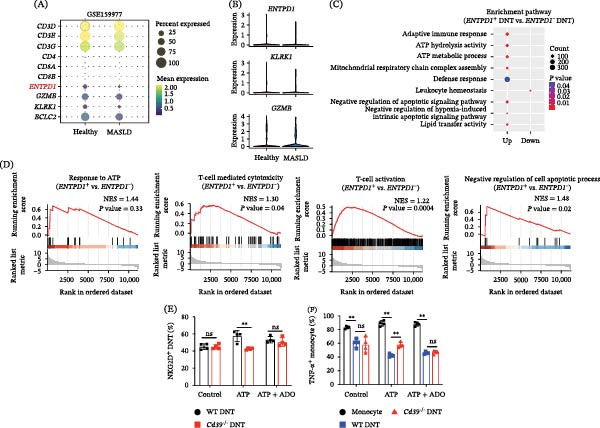
Downregulated CD39 expression in DNT cells impairs immunoregulation to promote MASLD. (A) Analysis of the single‐cell public dataset GSE159977 comparing expression differences of *ENTPD1* (CD39), *GZMB* (granzyme B), *KLRK1* (NKG2D), and *BCL2* in hepatic DNT cells (defined as *CD3D* ^*+*^ *CD3E* ^*+*^ *CD3G* ^*+*^ *CD4* ^*−*^ *CD8A* ^*−*^ *CD8B* ^*−*^ subset) between healthy controls and MASLD patients. (B) Violin plots showing the expression levels of *ENTPD1*, *KLRK1*, and *GZMB* in DNT cells. (C) DNT cells from MASLD patients in the GSE159977 dataset were stratified into *ENTPD1* ^+^ and *ENTPD1* ^−^ populations, followed by pathway enrichment analysis of DEGs between these subgroups (|fold change| ≥ 2, *p* < 0.05). (D) GSEA of *ENTPD1* ^*+*^ versus *ENTPD1* ^*−*^ DNT cells. (E) Flow cytometric analysis of NKG2D expression in wild‐type (WT) or *Cd39* ^−/−^ DNT cells after in vitro stimulation with ATP or ATP plus adenosine. Actual *p* values for panel E are as follows (left to right): 0.9468, 0.0035, and 0.4001. (F) WT or *Cd39* ^−/−^ DNT cells were cocultured with CD45.1^+^ macrophages for 6 hr in the presence of ATP or ATP plus adenosine, followed by measurement of TNF‐α secretion by macrophages. Actual *P* values for panel F are as follows (left to right): 0.0040, 0.8847, <0.0001, 0.0002, <0.0001, and 0.7370. Error bars represent mean ± SD. *n* = 4 biologically independent samples per group. Statistical analysis was performed using a multiple unpaired *t*‐test for panel E, and one‐way ANOVA followed by Sidak’s test for panel F.  ^∗^ *p* < 0.05,  ^∗∗^ *p* < 0.01.

To investigate the CD39 expression levels in hepatic DNT cells from MASLD patients, we analyzed single‐cell RNA sequencing (scRNA‐seq) data from liver CD45^+^ immune cells (GEO: GSE159977). DNT cells were defined as *CD3D* ^+^ *CD3E* ^+^ *CD3G* ^+^ with the concomitant absence of *CD4*, *CD8A*, and *CD8B*. *ENTPD1* (encoding CD39) expression was downregulated in DNT cells from MASLD patients, with a parallel reduction in the expression of *GZMB* (granzyme B) and *KLRK1* (NKG2D) and the anti‐apoptotic gene *BCL2* (Figure 2A). Further Violin plot analysis revealed that the decrease in *KLRK1* expression was more pronounced than that of *GZMB* (Figure 2B), suggesting that CD39 downregulation predominantly affects NKG2D‐related functions in DNT cells. To further investigate the regulatory role of CD39 in DNT cells, we stratified DNT cells from MASLD patients in the database into *ENTPD1* ^+^ and *ENTPD1* ^−^ subsets and compared their functional signatures. Pathway enrichment and GSEA revealed that, compared with their *ENTPD1* ^+^ counterparts, *ENTPD1* ^−^ DNT cells exhibited reduced ATP hydrolysis activity, impaired T‐cell cytotoxicity and activation, and increased apoptotic signaling (Figure 2C,D).

In this study, DNT cell immunoregulatory activity was evaluated by their NKG2D‐associated suppression of proinflammatory macrophage activation, with macrophage TNF‐α production as a key functional readout. To directly validate the functional significance of CD39 in DNT cell immunoregulation, we further performed complementary functional experiments using WT and *Cd39* knockout (*Cd39* ^*−/−*^) DNT cells. Given the central role of CD39 as an ATP‐hydrolyzing enzyme, DNT cells were cultured under control conditions, with ATP alone or with ATP plus adenosine, and NKG2D expression as well as the capacity to suppress macrophage activation was assessed. Under conditions without ATP, WT and *Cd39* ^*−/−*^ DNT cells displayed comparable NKG2D expression and similar abilities to suppress macrophage TNF‐α production. In the presence of ATP, WT DNT cells generated adenosine through CD39‐mediated ATP hydrolysis, thereby maintaining a higher NKG2D expression and more effectively suppressing macrophage activation. In contrast, under ATP stimulation, *Cd39* ^*−/−*^ DNT cells were unable to generate adenosine, resulting in lower NKG2D expression and impaired immunosuppressive capacity relative to WT DNT cells. Importantly, supplementation with exogenous adenosine restored NKG2D expression and macrophage‐suppressive function in both WT and *Cd39* ^*−/−*^ DNT cells, abolishing the difference between the two groups (Figure 2E,F). These results are consistent with a CD39‐dependent pathway in which ATP hydrolysis supports DNT cell survival and NKG2D‐associated immunoregulatory activity.

### 3.3. AA Downregulates CD39 Expression in DNT Cells

**Figure 3:**
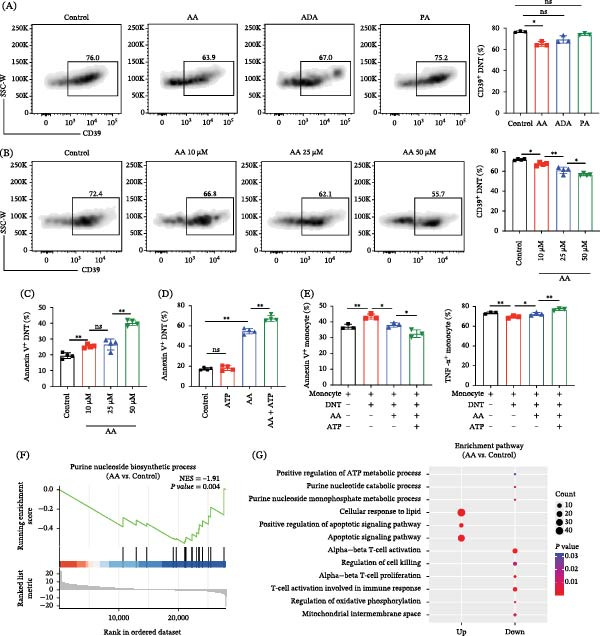
Arachidonic acid downregulates CD39 expression in DNT cells. (A) DNT cells were stimulated with arachidonic acid (AA, 25 µM), adrenic acid (ADA, 25 µM), or palmitic acid (PA, 25 µM) for 48 h, followed by flow cytometry analysis of CD39 expression levels. Actual *p* values for panel A are as follows (left to right): 0.0137, 0.0809, and 0.8437. *n* = 3 biologically independent samples per group. (B, C) DNT cells were treated with AA (10, 25, or 50 µM) for 48 h, and CD39 expression and apoptotic levels were detected via flow cytometry. Actual *p* values for panels B and C are as follows (left to right): panel B—0.0197, 0.0017, and 0.0197; panel C—0.0074, 0.8722, and <0.0001. *n* = 4 biologically independent samples per group. (D) DNT cells were treated with ATP (400 µM), AA (25 µM), or ATP + AA for 48 h, and apoptosis was assessed. Actual *p* values for panel D are as follows (left to right): 0.9575, <0.0001, and <0.0001. *n* = 4 biologically independent samples per group. (E) In vitro, DNT cells pretreated with AA (25 µM) for 48 h were cocultured with CD45.1^+^ monocytes at a 1:2 ratio in medium containing 400 µM ATP for 6 h, followed by evaluation of CD45.1^+^ monocyte apoptosis and TNF‐α secretion. Actual *p* values for panel E are as follows (left to right): 0.0071, 0.0202, 0.0161, 0.0091, 0.0455, and 0.0057. *n* = 3 biologically independent samples per group. (F) Transcriptomic sequencing data from AA‐treated (25 µM) DNT cells and untreated controls were subjected to GSEA. *n* = 3 biologically independent mice per group. (G) Enriched pathway analysis was conducted using DEGs (|fold change| ≥ 2, *p* < 0.05) from AA‐treated versus control DNT cells. Error bars represent mean ± SD. Statistical analysis was performed using the Kruskal–Wallis test with Dunn’s test for panel A, and one‐way ANOVA followed by Sidak’s test for panels B–E.  ^∗^ *p* < 0.05,  ^∗∗^ *p* < 0.01.

Lipotoxicity mediated by hepatic fatty acid accumulation is a central driver of MASLD pathogenesis [10]. Our prior metabolomic profiling revealed elevated hepatic levels of harmful fatty acids, including AA, ADA, and pimelic acid (PA), in MASLD model mice [5]. Importantly, CD39 expression in DNT cells was dose‐dependently downregulated by AA stimulation but remained unaffected by ADA or PA (Figure 3A,B), indicating that AA is a principal mediator of downregulating CD39 expression in MASLD‐associated DNT cell dysfunction.

Since CD39 functions as an ATPase that hydrolyzes extracellular ATP, we assessed DNT cell viability and function under AA and ATP challenges. AA stimulation significantly promoted DNT cell apoptosis, and this effect became more pronounced with increasing concentrations of AA or additional supplementation with ATP (Figure 3C,D). In vitro coculture experiments revealed that AA stimulation reduced the immunoregulatory function of DNT cells in suppressing monocyte survival and TNF‐α secretion. Additionally, the addition of ATP to AA‐stimulated cells further decreased the immunosuppressive function of DNT cells (Figure 3E). These findings establish that AA downregulates CD39 expression in DNT cells, thereby compromising their viability and immunoregulatory capacity.

Moreover, the effect of AA on DNT cells was explored through transcriptome analysis of AA‐treated DNT cells, as reported in our previous study [5]. As expected, GSEA and enrichment pathway analysis revealed that AA stimulation upregulated the expression of the gene sets associated with the cellular response to lipid and apoptotic signaling pathways but significantly downregulated the expression of the gene sets related to T‐cell activation and cell killing. Furthermore, AA stimulation significantly inhibited the purine nucleoside biosynthetic process and the purine nucleotide catabolic process in DNT cells (Figure 3F,G), confirming that AA is a key factor contributing to the reduced CD39 expression and impaired immunoregulation in DNT cells.

### 3.4. AA Suppresses CD39 Expression via HIF‐1α

**Figure 4:**
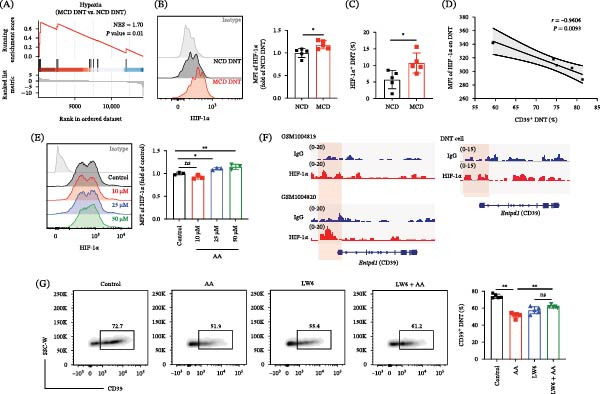
Arachidonic acid suppresses CD39 expression via HIF‐1α. (A) GSEA was performed on hepatic DNT cells isolated from MCD‐fed MASLD mice versus NCD‐fed control mice. *n* = 2 biologically independent mice per group. (B, C) Flow cytometry analysis of HIF‐1α MFI and HIF‐1α^+^ DNT cell percentage in the liver of MCD‐fed and NCD‐fed mice. Actual *p* values for panels B and C are 0.0178 and 0.0263, respectively. *n* = 5 biologically independent mice per group. (D) Correlation analysis between HIF‐1α expression and CD39 level in hepatic DNT cells from MCD‐fed mice. Actual *p* values for panels D is 0.0093. *n* = 5 biologically independent mice per group. (E) In vitro, DNT cells were stimulated with AA (10, 25, or 50 µM) for 48 h, followed by flow cytometric quantification of HIF‐1α expression. Actual *p* values for panel E are as follows (left to right): 0.1238, 0.0410, and 0.0061. *n* = 3 biologically independent samples per group. (F) ChIP‐seq and CUT&Tag analysis showed HIF‐1α binding at the *Entpd1* promoter region (encoding CD39). (G) DNT cells were pretreated with the HIF‐1α inhibitor LW6 (10 µM) for 1 h prior to AA (25 µM) stimulation for 48 h, and CD39 expression was analyzed by flow cytometry. Actual *p* values for panel G are as follows (left to right): <0.0001, 0.0001, and 0.1029. *n* = 5 biologically independent samples per group. Error bars represent mean ± SD. Statistical analysis was performed using an unpaired two‐tailed Student’s *t*‐test for panels B and C, Pearson correlation analysis for panel D, and one‐way ANOVA followed by Sidak’s multiple comparisons test for panels E and G.  ^∗^ *p* < 0.05,  ^∗∗^ *p* < 0.01.

The HIF‐1α subunit is a transcription factor that suppresses the expression of CD39 in T cells [11]. Compared with NCD‐fed control mice, MCD diet‐induced MASLD model mice presented significant hypoxia‐related signaling pathway activation in hepatic DNT cells (Figure 4A). Flow cytometry analysis confirmed elevated HIF‐1α protein levels in hepatic DNT cells from MCD‐fed mice (Figure 4B,C), with HIF‐1a expression inversely correlated with CD39 expression in these cells (Figure 4D). On the basis of these findings, we hypothesized that AA induces the downregulation of CD39 expression in DNT cells via HIF‐1α. In support of this hypothesis, AA stimulation dose‐dependently elevated HIF‐1α protein levels in DNT cells (Figure 4E).

To examine the direct transcriptional regulation of CD39 by HIF‐1α, we analyzed chromatin immunoprecipitation sequencing (ChIP‐seq) data for HIF‐1α in T cells (GSM1004819 and GSM1004820). Analysis of the HIF‐1α‐bound regions revealed marked enrichment at the promoter region of *Entpd1* (encoding CD39), providing further evidence that HIF‐1α directly regulates CD39 gene expression. Consistently, analysis of our previously published HIF‐1α CUT&Tag data in DNT cells [12] revealed enrichment at the *Entpd1* promoter (Figure 4F). Importantly, pharmacological inhibition of HIF‐1α with LW6 abrogated the AA‐induced downregulation of CD39 in DNT cells, confirming that the regulatory effect of AA on CD39 expression is mediated through the HIF‐1α pathway (Figure 4G). These results demonstrate that AA stimulation induces HIF‐1α–mediated transcriptional repression of *Entpd1* (encoding CD39), thereby impairing the hydrolysis of extracellular ATP to adenosine.

### 3.5. *In Vivo* Inhibition of HIF‐1α Modulates Hepatic DNT Cells and Ameliorates MASLD

**Figure 5:**
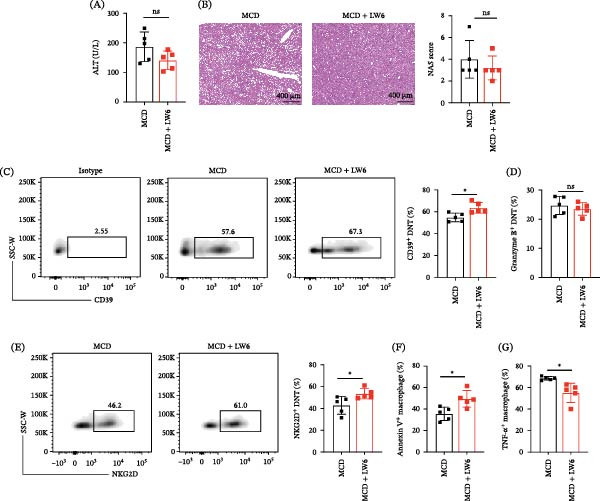
In vivo inhibition of HIF‐1α modulates hepatic DNT cells and ameliorates MASLD. (A) MASLD was induced by feeding mice an MCD diet, followed by treatment with the HIF‐1α inhibitor LW6 (10 mg/kg, intraperitoneally, every other day) after 14 days of MCD feeding. Serum ALT levels were measured. (B) Representative H&E staining of liver sections and quantification of NAS scores. (C–E) Flow cytometric analysis of CD39, granzyme B, and NKG2D expression in hepatic DNT cells. (F, G) Assessment of hepatic macrophage apoptosis and TNF‐α production. Actual *p* values from panels A–G are as follows: 0.1195, 0.4841, 0.0134, 0.4994, 0.0343, 0.0136, and 0.0107. Error bars represent mean ± SD. *n* = 5 biologically independent mice per group. Statistical analyses were performed using a Mann–Whitney *U* test for panel B and an unpaired two‐tailed Student’s *t*‐test for all other panels.  ^∗^ *p* < 0.05,  ^∗∗^ *p* < 0.01.

To further validate the functional relevance of the HIF‐1α–CD39 axis in vivo, we examined the effects of pharmacological HIF‐1α inhibition in a MASLD mouse model. MASLD was induced by feeding mice an MCD diet, followed by treatment with the HIF‐1α inhibitor LW6 (10 mg/kg, intraperitoneally, every other day) after 14 days of MCD feeding. LW6 treatment reduced serum ALT levels and partially attenuated hepatic steatosis in H&E staining (Figure 5A,B).

LW6 increased the expression of CD39 and, concomitantly, the expression of NKG2D in hepatic DNT cells, while granzyme B expression remained unchanged (Figure 5C–E). In parallel, LW6‐treated mice exhibited increased apoptosis of hepatic macrophages and reduced hepatic TNF‐α production, indicating an overall attenuation of hepatic inflammatory responses (Figure 5F,G; the gating strategy is shown in Figure S1). Together, these in vivo data indicate that HIF‐1α inhibition modulates the immunoregulatory phenotype of hepatic DNT cells, but this immunomodulation alone was not sufficient to translate into measurable improvement of overall MASLD pathology, consistent with the multifactorial pathogenesis of the disease.

## 4. Discussion

Immunometabolic dysregulation is central to the pathogenesis of MASLD. Excessive free fatty acid‐induced lipotoxicity contributes to hepatic mitochondrial dysfunction and endoplasmic reticulum stress, triggering an inflammatory cascade and immune cell activation that further damages stressed hepatocytes, thereby perpetuating a vicious cycle [13]. In this study, we demonstrated that DNT cells, an immunoregulatory T‐cell subset critical for hepatic immune balance, lose their viability and immunoregulatory function under MASLD conditions. This impairment stems from lipotoxicity mediated by AA, which reduces CD39 expression via HIF‐1α. The downregulation of CD39 expression disrupts purinergic signaling, suppressing DNT cell survival and weakening DNT cell ability to suppress inflammation, thereby accelerating MASLD development.

CD39, an ectonucleotidase that hydrolyzes extracellular ATP to adenosine, is pivotal for immune regulation. In conjunction with CD73, CD39 establishes an anti‐inflammatory microenvironment by generating adenosine, which suppresses effector immune responses. CD39 serves as a distinct functional marker for Tregs. In contrast to CD25^hi^ Tregs, CD39^hi^ Tregs exhibit sustained FOXP3 expression and immunosuppressive activity, establishing CD39 as a specific marker for identifying functionally stable Treg subsets. Mechanistically, adenosine generated through CD39/CD73‐mediated ATP hydrolysis engages the A2A receptor (A2AR) on Tregs, activating the CEBPβ‐dependent pathway to drive IL‐10 production and reinforce immunosuppression [14]. Our prior work revealed that, in addition to granzyme B and perforin, CD39 acts as a key effector molecule in DNT cells, enhancing their activity and function through adenosine production [8]. In this study, we observed reduced CD39 in hepatic DNT cells from MASLD mice. In vitro, under ATP stimulation, CD39 supported the maintenance of the NKG2D expression and promoted the capacity of DNT cells to restrain macrophage activation. Consistently, human liver scRNA‐seq data showed reduced *ENTPD1* and functional markers in DNT cells from MASLD patients. However, the functional relevance of CD39 in human hepatic DNT cells and the mechanisms driving its downregulation in MASLD remain to be further defined.

Lipids and lipid metabolism are critical regulators of T‐cell function. For example, oleic acid enhances Treg‐mediated suppression by stabilizing FOXP3 and promoting the fatty acid oxidation–oxidative phosphorylation metabolic program [15]. In contrast, a high content of AA promotes a population of vulnerable Tregs that are prone to lipid peroxidation and ferroptosis, ultimately leading to a reduced suppressive capacity [16]. Similarly, our data show that AA, which is enriched in MASLD livers, reduces CD39 expression in DNT cells in a HIF‐1α dependent manner, thereby weakening the ability of DNT cells to restrain monocyte survival and TNF‐α production. Of note, the preferential effect of AA compared with ADA and PA may reflect its rapid conversion by COX, LOX, and CYP450 pathways into a broad range of bioactive oxidized lipid mediators that can engage inflammatory signaling and intersect with hypoxia‐related pathways [17, 18]. In contrast, ADA can also yield oxidized lipids but may generate a distinct mediator profile, whereas PA is a saturated fatty acid that is more closely linked to lipotoxic stress responses such as endoplasmic reticulum stress and mitochondrial dysfunction [19, 20]. Further studies will be needed to determine the upstream metabolites and pathways underlying this preference and to define how they connect AA to HIF‐1α dependent regulation of CD39 in DNT cells. These findings reveal a lipid‐specific mechanism by which AA disrupts DNT cell‐mediated immunoregulation.

Lipid overload exacerbates hepatic hypoxia in MASLD through structural and hemodynamic alterations. Ballooned hepatocytes compress the space of Disse, impairing sinusoidal blood flow and thereby diminishing the oxygen supply to hepatocytes [21]. This hypoxic microenvironment promotes the nuclear translocation of hypoxia‐inducible factors (HIF‐1α/HIF‐2α). HIF‐1α, a key transcriptional regulator of CD39 in T cells, disrupts aryl hydrocarbon receptor (AHR) signaling and impairs CD39 expression, whereas inhibition of HIF‐1a restores CD39 expression in T cells [11, 22]. Notably, our study revealed elevated HIF‐1α levels in MASLD‐derived DNT cells, an effect that was inversely correlated with the CD39 expression. Mechanistically, AA treatment directly upregulated HIF‐1α in DNT cells, establishing a direct link between lipid overload and downregulated CD39 expression. Further ChIP‐seq analysis and our HIF‐1α CUT&Tag data indicated HIF‐1α enrichment at the *Entpd1* promoter, supporting that HIF‐1α can directly regulate CD39 transcription in DNT cells. Consistently, inhibition of HIF‐1α with LW6 prevented the AA‐induced downregulation of CD39 in DNT cells in vitro and increased CD39 expression in hepatic DNT cells in the MASLD mouse model, supporting a role for HIF‐1α signaling in the regulation of CD39.

In summary, this study identified a lipid‐purinergic signaling axis that disrupts DNT cell function in MASLD. AA, through HIF‐1α, suppresses CD39 expression in DNT cells, impairing their survival and immunoregulatory capacity. These findings highlight the therapeutic potential of targeting metabolic‒immune crosstalk to restore hepatic homeostasis in MASLD.

## Author Contributions

**Hua Jin**: investigation, data curation, funding acquisition, writing – original draft. **Xiyu Wang**: investigation, data curation, writing – original draft. **Xinjuan Liu**: writing – review and editing. **Dong Zhang**: conceptualization, funding acquisition, writing – review and editing. **Guangyong Sun**: conceptualization, supervision, funding acquisition, writing – review and editing.

## Funding

This study was supported by the National Natural Science Foundation of China (Grants 82202021, 82270606, and 82370578).

## Conflicts of Interest

The authors declare no conflicts of interest.

## Supporting Information

Additional supporting information can be found online in the Supporting Information section.

## Supporting information


**Supporting Information** Figure S1: Representative flow cytometry gating strategy for hepatic monocyte‐derived macrophages (CD45+Ly6G‐F4/80intCD11blow).

## Data Availability

Data sharing is not applicable to this article as no datasets were generated or analyzed during the current study.
