# Supplementary material for: Hepatic Arachidonic Acid Dysregulation Impairs Double‐Negative Regulatory T Cell‐Mediated Immunoregulation via CD39 Downregulation and Drives MASLD Development
Source: J Immunol Res. 2026 Jun 25;2026:6950401. doi: 10.1155/jimr/6950401 (PMC13296590; doi:10.1155/jimr/6950401)
Supplement: Supplementary file 1 — Supporting Information Figure S1: Representative flow cytometry gating strategy for hepatic monocyte‐derived macrophages (CD45+Ly6G‐F4/80intCD11blow). [file JIMR-2026-6950401-s001.docx]

**Supplementary Figure**

Figure S1. Representative flow cytometry gating strategy for hepatic monocyte-derived macrophages (CD45^+^Ly6G^-^F4/80^int^CD11b^low^).
